# Photoredox Coupling of CO_2_ Reduction with Benzyl Alcohol Oxidation over Ternary Metal Chalcogenides (Zn_m_In_2_S_3+m_, m = 1–5) with Regulable Products Selectivity

**DOI:** 10.3390/molecules28186553

**Published:** 2023-09-10

**Authors:** Zisheng Du, Kexin Gong, Zhiruo Yu, Yang Yang, Peixian Wang, Xiuzhen Zheng, Zhongliao Wang, Sujuan Zhang, Shifu Chen, Sugang Meng

**Affiliations:** 1 Key Laboratory of Green and Precise Synthetic Chemistry and Applications, Ministry of Education, College of Chemistry and Materials Science, Huaibei Normal University, Huaibei 235000, China; 2 Key Laboratory of Pollutant Sensitive Materials and Environmental Remediation, Key Laboratory of Clean Energy and Green Circulation, Huaibei Normal University, Huaibei 235000, China; 3 Shanghai Key Laboratory of Atmospheric Particle Pollution and Prevention (LAP3), Fudan University, Shanghai 200438, China; 4 State Key Laboratory Incubation Base for Green Processing of Chemical Engineering, School of Chemistry and Chemical Engineering, Shihezi 832003, China

**Keywords:** photocatalytic, CO_2_ reduction, syngas, benzyl alcohol oxidation, ZnIn sulfide

## Abstract

Integrating photocatalytic CO_2_ reduction with selective benzyl alcohol (BA) oxidation in one photoredox reaction system is a promising way for the simultaneous utilization of photogenerated electrons and holes. Herein, Zn_m_In_2_S_3+m_ (m = 1–5) semiconductors (ZnIn_2_S_4_, Zn_2_In_2_S_5_, Zn_3_In_2_S_6_, Zn_4_In_2_S_7_, and Zn_5_In_2_S_8_) with various composition faults were synthesized via a simple hydrothermal method and used for effective selective dehydrocoupling of benzyl alcohol into high-value C–C coupling products and reduction of CO_2_ into syngas under visible light. The absorption edge of Zn_m_In_2_S_3+m_ samples shifted to shorter wavelengths as the atomic ratio of Zn/In was increased. The conduction band and valence band position can be adjusted by changing the Zn/In ratio, resulting in controllable photoredox ability for selective BA oxidation and CO_2_ reduction. For example, the selectivity of benzaldehyde (BAD) product was reduced from 76% (ZnIn_2_S_4_, ZIS1) to 27% (Zn_4_In_2_S_7_, ZIS4), while the selectivity of hydrobenzoin (HB) was increased from 22% to 56%. Additionally, the H_2_ formation rate on ZIS1 (1.6 mmol/g/h) was 1.6 times higher than that of ZIS4 (1.0 mmol/g/h), and the CO formation rate on ZIS4 (0.32 mmol/g/h) was three times higher than that of ZIS1 (0.13 mmol/g/h), demonstrating that syngas with different H_2_/CO ratios can be obtained by controlling the Zn/In ratio in Zn_m_In_2_S_3+m_. This study provides new insights into unveiling the relationship of structure–property of Zn_m_In_2_S_3+m_ layered crystals, which are valuable for implementation in a wide range of environment and energy applications.

## 1. Introduction

With the combustion of fossil fuels, a large amount of carbon dioxide (CO_2_) is emitted into the air, leading to significant changes in both the environment and energy dynamics [1,2]. Solar-powered conversion of CO_2_ into valuable fuels or feedstock has been recognized as a sustainable and environmentally friendly energy conversion technology to address these problems [3,4,5,6,7,8]. This approach is considered a win–win strategy as it can effectively reduce the greenhouse effect while also alleviating the pressure of energy scarcity. However, the conversion efficiencies of CO_2_ are currently unsatisfactory due to the stable structure of CO_2_. Additionally, typical photocatalytic systems are performed in water, which results in low evolution efficiency of O_2_ due to the large overpotential [9]. Most CO_2_ photoreduction studies focus on the reductive half-reaction, with less attention being paid to the oxidative half-reaction [10,11]. The use of sacrificial reagents such as isopropyl alcohol (IPA), triethanolamine (TEOA), sulfite, etc., to capture holes can accelerate the reaction rate but produces less value oxidation products [12,13,14,15,16]. Merging photocatalytic CO_2_ reduction with organic synthesis into one system may be an ideal strategy [1,13]. Allowing the holes to react with organic substrates instead of H_2_O or hole scavengers can facilitate the production of value-added chemicals and improve CO_2_ reduction efficiency.

Among the organic substrates, benzyl alcohol (BA) is one of the most popular because it can be oxidized to value-added chemicals such as benzaldehyde (BAD) and C-C coupling products, including benzoin, deoxybenzoin, and hydrobenzoin (HB), which are widely used as versatile structural motifs in fine chemicals and pharmaceutical intermediates [17,18,19]. Metal sulfide semiconductors, known for their high redox ability, good visible-light responses, and rich variability in properties, have been widely used in photocatalytic fields, including CO_2_ reduction, H_2_ evolution, and BA oxidation, etc. [15,19,20,21]. Nevertheless, the majority of semiconductors demonstrate inadequate photocatalytic activities due to their limited light absorption properties and inefficient charge separation. To achieve the goal of industrial application, scientists have been actively researching catalyst modification techniques such as morphology control, defect engineering, heterojunction construction, and co-catalyst loading to overcome challenges related to slow electron transport behavior, high carrier recombination efficiency, and to effectively optimize the interface structure and behavior of catalysts, ultimately improving catalytic efficiency and selectivity. For example, Han et al. designed controllable Au–Pt@CdS hybrids for photoredox conversion of alcohol to valuable aldehyde and H_2_ [22]. Qi et al. reported SiO_2_-supported semiconductor CdS quantum dots, which exhibited high efficiency in dehydrogenative C−C coupling of BA into C−C coupled HB with high selectivity (95−100%) [17]. Kevin et al. prepared CdS QDs to achieve visible light-driven oxidation of BA with >90% selectivity for either BAD or C−C coupled products (including deoxybenzoin, benzil, and HB), by tuning the amount of Cd^0^ deposited on the CdS QD surfaces in situ [23]. Intriguingly, ZnIn sulfide with a customized Zn/In ratio and controllable band structure has attracted significant attention [20]. The former work has demonstrated that the coproduction of C−C coupled products and hydrogen (H_2_) can be controlled by altering the Zn/In ratio of Zn_x_In_2_S_3+x_ (x = 0.1, 0.2, 0.4, 0.6, and 0.8); however, the selectivity of specific C−C coupled product is very low [24]. The photocatalytic activities of Zn_x_In_2_S_3+x_ (x = 1–5) were explored for photocatalytic hydrogen production from water and CO_2_ reduction [25]. Additionally, the Zn_x_In_2_S_3+x_ photocatalyst also showed high performances for lignin depolymerization to functionalized aromatics [26]. Previous studies have proven that doping, constructing heterojunction, or optimizing the atomic ratio of Zn and In can significantly enhance photocatalytic performance. However, the optimization of the atomic ratio offers a unique approach to improving photocatalytic performance. This is because by controlling the presence and distribution of composition faults, which disrupt the crystal structure’s periodicity, the atomic ratio directly influences charge carrier dynamics and interfacial chemical reactions. Furthermore, the resulting anisotropic electrical conductivity from these composition faults promotes efficient charge separation and transfer, ultimately leading to improved photocatalytic activity. Unfortunately, not much attention has been given to the effects of composition faults of Zn_x_In_2_S_3+x_ layered crystals in the field of simultaneous photocatalytic CO_2_ reduction and selective BA oxidation. This work aims to reveal the relationship between the structure and properties of Zn_m_In_2_S_3+m_ in CO_2_ reduction and BA oxidation reactions in one system.

In this study, a series of ZnIn sulfides (Zn_m_In_2_S_3+m_ (m = 1–5, integer)) with various Zn/In ratios were synthesized via a simple hydrothermal method. ZnIn_2_S_4_, Zn_2_In_2_S_5_, Zn_3_In_2_S_6_, Zn_4_In_2_S_7_, and Zn_5_In_2_S_8_ were defined as ZIS1, ZIS2, ZIS3, ZIS4, and ZIS5, respectively. Their structure information and typical physicochemical properties were characterized by various characterization techniques, such as scanning electron microscopy (SEM), transmission electron microscopy (TEM), X-ray diffraction (XRD) spectra, UV-visible diffuse reflectance spectroscopy (UV–vis DRS), X-ray photoelectron spectroscopy (XPS), and so on. The relationship between structure and photogenerated charges were investigated by photocurrent, electrochemical impedance spectroscopy (EIS), and photoluminescence spectra, etc. Also, gas chromatography (GC) and high-performance liquid chromatography (HPLC) were employed to probe the products’ composition, such as H_2_, CO, BZ, BAD, HB, etc. Additionally, in situ diffuse reflectance infrared Fourier transform spectroscopy (DRIFTS) and density functional theory (DFT) calculation were carried out to explore the reaction mechanism on catalysts with different Zn/In ratios. The results showed that the band structure, H_2_/CO ratio and selectivity of oxidation products of BA could be regulated by altering the Zn/In ratio. By conducting a thorough analysis of the photoproducts of H_2_, CO, BZ, BAD, and HB, we aim to uncover important trends and insights that can contribute to the development of more efficient catalysts for these reactions.

## 2. Results and Discussion

### 2.1. Characterization of Catalysts

**Figure 1:**
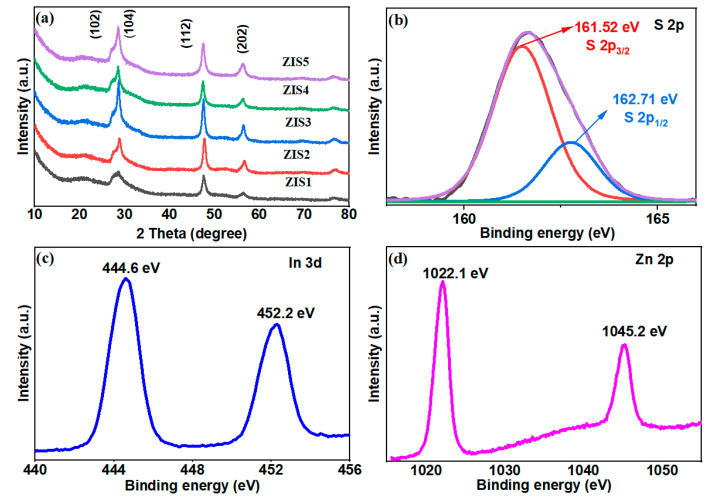
(**a**) XRD patterns of ZIS samples and high-resolution XPS spectra of (**b**) S 2p, (**c**) In 3d, (**d**) Zn 2p of ZIS4.

The XRD patterns of Zn_m_In_2_S_3+m_ (m = 1–5) composites showed similar patterns as shown in Figure 1. The peaks at 21.6, 26.8, 28.1, 47.0, 52.2, 55.9, and 76.8° can be assigned to (006), (102), (104), (112), (1012), (202), and (213) facets, respectively, which can be attributed to the hexagonal phase [26,27,28]. Oxides, binary sulfides, or organic compounds related to the reactants were not detected via the XRD analysis, indicating the prepared Zn_m_In_2_S_3+m_ (m = 1–5) samples were relatively pure, which was consistent with the reports [26,27]. It was reported that the chemical compositions of Zn_m_In_2_S_3+m_ are different, and the structure characteristics are similar, as a result, showing similar XRD patterns [25,28].

The X-ray photoelectron spectroscopy (XPS) was further employed to study the surface composition and chemical state of the ZIS4 sample. Figure 1b shows the high-resolution spectra of S 2p, in which the peak located at 161.7 eV can be attributed to S^2−^. In Figure 1b, the deconvoluted S 2p XPS spectrum reveals distinct peaks at approximately 161.52 and 162.71 eV (with an energy difference of 1.19 eV), corresponding to S 2p3/2 and S 2p1/2, respectively [29,30]. The two characteristic peaks at approximately 444.6 and 452.2 eV correspond to In 3d_5/2_ and 3d_1/2_, respectively, demonstrating that the valence state of the indium was +3 [25,31]. The Zn 2p spectra of ZIS4 exhibit peaks around 1022.1 and 1045.2 eV, corresponding to the Zn 2p_3/2_ and 2p_1/2_ of Zn^2+^, respectively [32]. The XRD and XPS results demonstrated that layer-structured Zn_m_In_2_S_3+m_ (m = 1–5) crystals were successfully synthesized.

**Figure 2:**
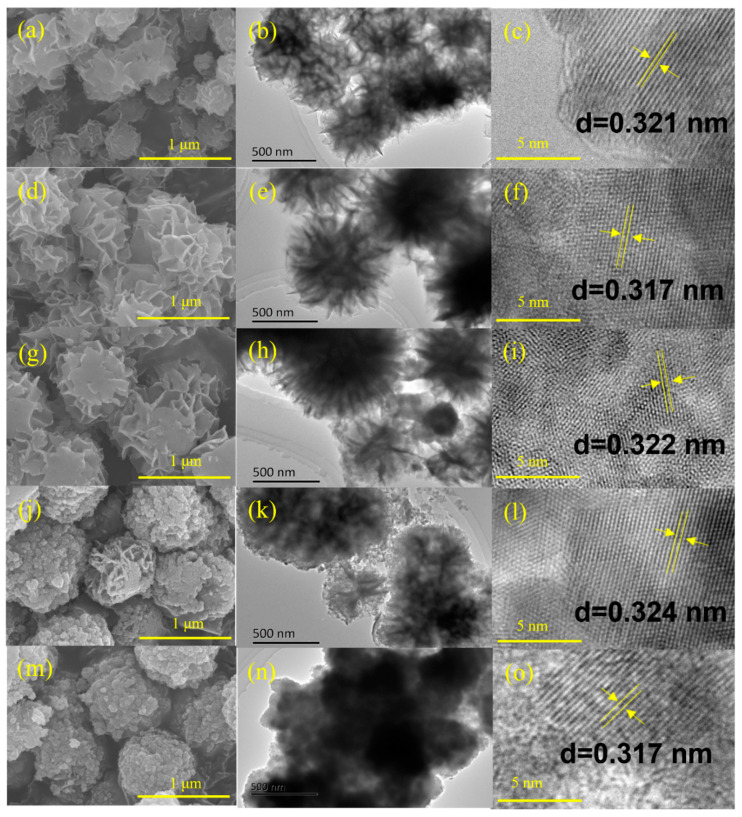
SEM, TEM, and HRTEM of ZIS1 (**a**–**c**), ZIS2 (**d**–**f**), ZIS3 (**g**–**i**), ZIS4 (**j**–**l**), and ZIS5 (**m**–**o**).

Figure 2 displays the morphology and structure of prepared Zn_m_In_2_S_3+m_ photocatalysts. The scanning electron microscopy (SEM) images (Figure 2a,d,g) show clearly that the ZnIn_2_S_4_, Zn_2_In_2_S_5_, and Zn_3_In_2_S_6_ samples were composed of cross-linked nanosheets and the average diameter of the microspheres was about 1 μm. Interestingly, the shapes of microspheres for Zn_4_In_2_S_7_ and Zn_5_In_2_S_8_ were partially distorted (shown in Figure 2j,m).

The transmission electron microscopy (TEM) images also display similar structures of Zn_m_In_2_S_3+m_ samples. With an increase of m, the sheets/petals that make up microspheres (Figure 2b) gradually become such small pieces (Figure 2n), which is consistent with SEM results. The high-resolution TEM (HRTEM) image of Zn_m_In_2_S_3+m_ catalysts are shown in Figure 2c,f,i,l,o. It is shown that the lattice fringes with an interplanar spacing of around 0.32 nm correspond to the (102) crystal plane of ZIS [25]. The XRD, XPS, SEM, as well as TEM results certified that all of the Zn_m_In_2_S_3+m_ (m = 1–5) layered crystals were successfully synthesized.

**Figure 3:**
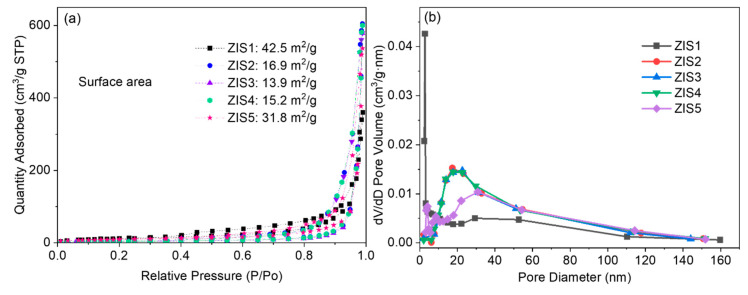
(**a**) Nitrogen adsorption-desorption isotherms and (**b**) pore-size distribution of ZIS samples.

The BET-specific surface area and pore-size distribution of the prepared samples were measured by nitrogen adsorption-desorption analysis (shown in Figure 3). The estimated surface areas of the ZIS1, ZIS2, ZIS3, ZIS4, and ZIS5 samples were 42.5, 16.9, 13.9, 15.2, and 31.8 m^2^/g, respectively. Additionally, the Zn_m_In_2_S_3+m_ samples also showed similar pore-size distributions, and the average pore diameters of ZIS1, ZIS2, ZIS3, ZIS4, and ZIS5 were about 52.3, 46.3, 47.3, 45.9, and 40.3 nm, respectively. A large pore size will facilitate effective transport pathways for product molecules and reactants [25]. The slightly decreased specific surface area may be caused by the distorted structure (SEM and TEM results).

**Figure 4:**
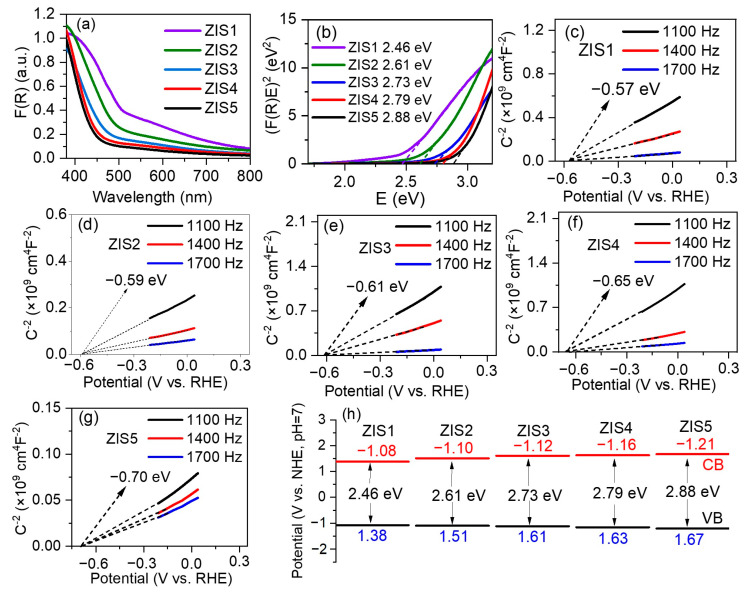
(**a**) UV-vis absorbance spectra, (**b**) Tauc plots, (**c**–**g**) Mott-Schottky plots, (**h**) energy band schematic diagram of typical catalysts.

The optical properties and energy band structure of Zn_m_In_2_S_m+3_ samples were studied using UV-vis diffuse reflectance spectroscopy (DRS) and Mott–Schottky measurements. As the value of m increased from 1 to 5, the absorption edges gradually shifted to shorter wavelengths (shown in Figure 4a), indicating an increase in band gap energy (Eg) of Zn_m_In_2_S_m+3_ samples with increasing Zn/In ratio. The Eg values of Zn_m_In_2_S_m+3_ were further calculated using the Kubelka–Munk function, [*F(R)hv*]^1/2^, plotted against the energy of light (Figure 4b) [26]. It was observed that the Eg of Zn_m_In_2_S_m+3_ increased from 2.46 to 2.88 eV with an increasing molar ratio of Zn to In. Specifically, band gap energies of ZnIn_2_S_4_, Zn_2_In_2_S_5_, Zn_3_In_2_S_6_, Zn_4_In_2_S_7_, and Zn_5_In_2_S_8_ are approximately 2.46, 2.61, 2.73, 2.79, and 2.88 eV, respectively. The color of the five typical catalysts are shown in Figure S1, and it was evident clearly that the color gradually faded with the increase in Zn/In ratio. Further, density functional theory (DFT) calculations were conducted to estimate the trend of band gap with changing the Zn/In ratio in ZIS, and the corresponding data were displayed in Figure S2. The calculated Eg of ZIS1 and ZIS4 was 0.143 and 0.367 eV, respectively, demonstrating an increase in the Eg of Zn_m_In_2_S_m+3_ with increasing molar ratio of Zn to In.

The flat-band potential (E_fb_) of the typical catalysts was further estimated by Mott–Schottky measurement. The Mott–Schottky curves indicated the samples exhibited n-type characteristics due to the positive slopes, and their flat-band potential was estimated to be −0.57, −0.59, −0.61, −0.65, −0.70 V vs. reversible hydrogen electrode (RHE), respectively (Figure 4c–g). The corresponding normal hydron electrode (NHE) potentials were calculated as −0.98, −1.00, −1.02, −1.06, −1.11 V vs. NHE. It has been reported that the conduction band minimum (CBM) of semiconductors is usually 0.1–0.2 V negative than the E_fb_ [33]. In this study, the CBMs of ZIS1, ZIS2, ZIS3, ZIS4, and ZIS5 were −1.08, −1.10, −1.12, −1.16, −1.21 V vs. NHE, respectively. According to Eg = E_VB_ − E_CB_, the E_VB_ of ZIS1, ZIS2, ZIS3, ZIS4, and ZIS5 were 1.38, 1.51, 1.61, 1.63, and 1.67 V vs. NHE (shown in Figure 4h), respectively. These results and trends are consistent with previous reports [25,26].

**Figure 5:**
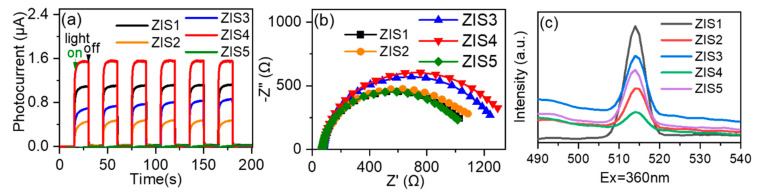
(**a**) Photocurrent density, (**b**) electrochemical impedance spectra (EIS), (**c**) steady-state photoluminescence spectra of typical catalysts.

The charge transfer behaviors are an important factor to explain the photocatalytic activity [19,34,35]. Photocurrent measurement was employed to assess the separation efficiency of photogenerated charge carries. As shown in Figure 5a, it is clear that the photocurrent increased sharply when the light was turned on and immediately returned to its initial negligible value when the light was switched off. ZIS4 exhibited the highest photocurrent, followed by ZIS1, ZIS3, ZIS2, and ZIS5 under light irradiation. Further, electrochemical impedance spectroscopy (EIS) was performed to study the charge transfer resistance of the typical photocatalysts [36,37]. From Figure 5b, it can be seen that ZIS1 has the smallest radii of the semicircles (nearly equal to ZIS5), implying that ZIS1 is beneficial for charge transfer. Photoluminescence (PL) spectra were also applied to investigate the transfer of photogenerated charge carriers [38,39]. In Figure 5c, ZIS4 exhibited the lowest PL intensity compared with other ZIS samples, meaning the ZIS4 had the lowest recommendation efficiency of electrons and holes; this was consistent with the photocurrent testing result. It can be inferred from the subsequent activity results that the product selectivity of both CO_2_ reduction and BA oxidation was not directly related to the BET-specific surface area and charge separation efficiency, which can be affected by various facets, including active sites, defects, composition, morphology, etc. [25].

### 2.2. Photoredox Reaction and Mechanism of CO_2_ Reduction with Oxidation of BA

**Figure 6:**
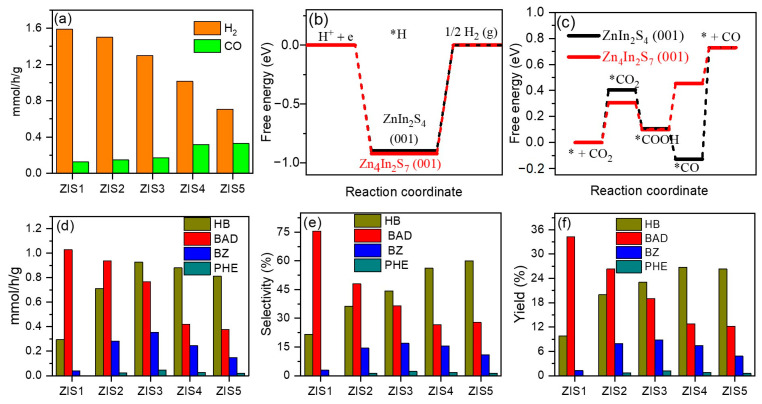
(**a**) Evolution rate of H_2_ and CO over ZIS samples, free energy for generating (**b**) H_2_ and (**c**) CO on ZIS1 and ZIS4 using DFT calculation, (**d**) generation rate, (**e**) selectivity, and (**f**) yield of products from BA oxidation over ZIS samples. “*” means the “active site”.

Subsequently, the photocatalytic CO_2_ reduction reaction integrated with selective oxidation of benzyl alcohol (BA) under the irradiation of visible light (λ > 420 nm) was studied using Zn_m_In_2_S_3+m_ samples (Figure 6). Figure 6a shows that ZIS1 exhibited the highest H_2_ formation rate of 1.6 mmol/g/h, followed by ZIS2, ZIS3, ZIS4, and ZIS5 at 1.5, 1.3, 1.0, and 0.7 mmol/g/h, respectively. While, the CO formation rate followed the order of ZIS5 (0.33 mmol/g/h) ≈ ZIS4 (0.32 mmol/g/h) > ZIS3 (0.17 mmol/g/h) > ZIS2 (0.15 mmol/g/h) > ZIS1 (0.13 mmol/g/h). This suggests that with increasing Zn content, the electrons preferentially react with CO_2_ rather than H^+^. The BET-specific surface area of the ZIS1 and ZIS4 samples was also measured using CO_2_ adsorption–desorption analysis (shown in Figure S3). The surface areas of the ZIS1 and ZIS4 samples were determined to be 41.03 and 44.89 m^2^/g, respectively, indicating that ZIS4 had a higher adsorption capacity of CO_2_. DFT calculations were performed to understand the critical role of Zn/In ratio in the selective photoreduction of CO_2_ to CO and H^+^ to H_2_ process over ZIS1 and ZIS4. The calculations revealed that the formation energy barrier of H* on ZIS1 and ZIS4 is 0.89 and 0.92 eV, respectively (Figure 6b), confirming that H_2_ formation is easier on ZIS1 than on ZIS4. Figure 6c shows that the formation energy barrier of *CO_2_ and *COOH on ZIS4 are lower than that of ZIS1, indicating that the CO_2_ reduction process is more favorable on the ZIS4 catalyst. These calculated results align well with the experimental findings.

Figure 6d displays that the formation rate of BAD was gradually decreased with increasing Zn/In ratio. On the other hand, the formation rates of the hydrogenation products, namely hydrogenation of benzyl alcohol (HB) and benzoin (BZ), increase with increasing Zn/In ratio and reach their highest values at ZIS3 (HB: 0.93 mmol/g/h and BZ: 0.35 mmol/g/h), after which they gradually decrease. Figure 6e shows that the selectivity of BAD was decreased from 76% (ZIS1) to 27% (ZIS4), while the selectivity of HB improved from 22% to 56% (ZIS4) and 60% (ZIS5). The yield of BAD also decreased from 34% (ZIS1) to 13% (ZIS4), while the yield of HB and BZ was increased from 10% and 1.2% to 27% and 8%, respectively (Figure 6f). These results demonstrated that both the H_2_/CO ratio and oxidation products of BA can be adjusted by altering the Zn/In raion in ZIS. The standard curves for BAD, HB, BE, and PHE are presented in Figure S4, while the HPLC spectra illustrating the products generated from ZIS1 and ZIS4 are depicted in Figure S5.

Furthermore, a series of control experiments were conducted to confirm the importance of both the CO_2_ atmosphere and BA substrate on the ZIS sample (shown in Figure S6). Without the addition of BA in the reaction system, the generation rates of H_2_ and CO were found to be negligible compared to those obtained when BA was present. Substituting BA with TEOA resulted in an improvement in the generation rate of H_2_, but a decrease in the generation rate of CO. When Ar was introduced into the cell instead of CO_2_, no CO was detected, and the produced H_2_ was also negligible. These findings suggest that CO is generated via CO_2_ reduction. However, the yield of BAD was determined as 64.5%, with a selectivity of 100% for BAD, and no other C-C coupling products were detected. These results demonstrate that both a CO_2_ atmosphere and a BA solution are essential for achieving a high generation rate of CO and C-C coupling products, such as HB and BZ.

**Figure 7:**
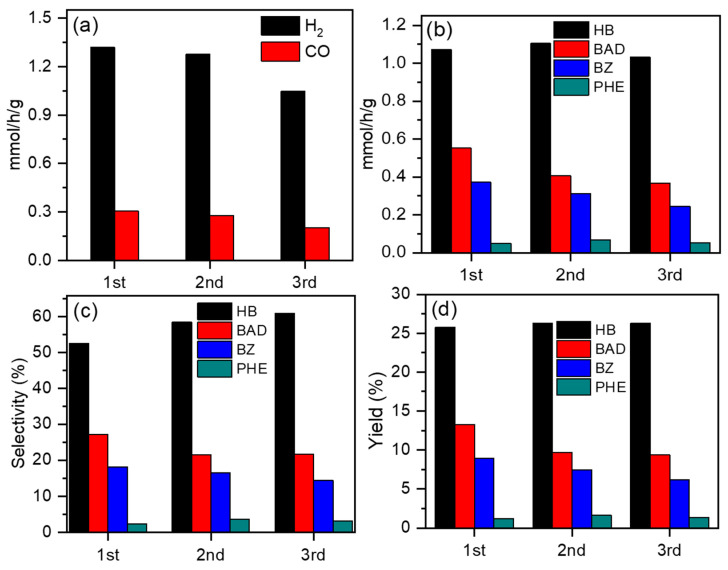
Photocatalytic CO_2_ reduction and BA oxidation performances on the ZIS4 sample under visible-light irradiation over three cycles. (**a**) The generation rate of H_2_ and CO, (**b**) generation rate, (**c**) selectivity, and (**d**) yield of products from BA oxidation.

To evaluate the stability of ZIS, ZIS4 was selected as the typical catalyst for testing. The catalytic stability of the ZIS4 sample was assessed through photocatalytic reusability analysis for three cycles, as shown in Figure 7. Figure 7a demonstrates that the H_2_ evolution rates were 1.31, 1.28, and 1.05 mmol/g/h in the 1st, 2nd, and 3rd runs, respectively. The generation rate of CO was 0.30, 0.27, and 0.21 mmol/g/h in the 1st, 2nd, and 3rd runs, respectively. The corresponding liquid products are displayed in Figure 7b. The formation rate of HB and PHE did not significantly decrease, while the generation rate of BAD and BZ slightly decreased. Figure 7c,d reveals that even after three cycles, the selectivity and yield of liquid products remained close to those of the first cycle. Furthermore, XRD profiles and SEM images (Figures S7 and S8) of ZIS4 before and after long-time experiments confirmed the well-maintained crystalline phase and morphology. The preservation of the crystalline structure and negligible loss of gas and liquid generation rates over three runs indicate the durability of the ZIS4 sample, making it a promising candidate for potential applications in sustainable energy conversion.

**Figure 8:**
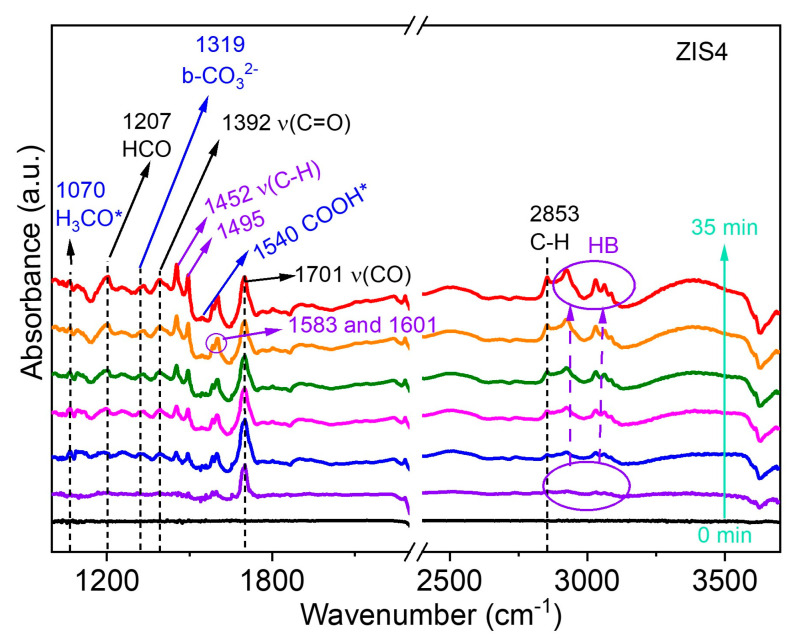
In situ DRIFTS spectra of the reaction intermediates in CO_2_ photoreduction and BA oxidation over ZIS4 under light irradiation. “*” means the “active site”.

Furthermore, the photocatalytic CO_2_RR and BA oxidation catalyzed by the ZIS4 catalyst at room temperature were investigated using in situ diffuse reflectance infrared Fourier transform spectroscopy (DRIFTS). The corresponding data can be found in Figure 8. The reaction intermediates were determined by analyzing the observed peaks in the infrared spectra. The peaks at 1319 cm^−1^ were attributed to the presence of bidentate carbonate (b-CO_3_^2−^) [3]. Additionally, the peaks at 1540 cm^−1^ were assigned to the COOH* group, which serves as an important intermediate for CO formation [3]. The infrared spectra also revealed the reaction intermediates involved in the conversion of BA. The peak at 1207 cm^−1^ was assigned to the presence of the formyl group (HCO) in BAD, while the sharp peak at 1701 cm^−1^ was attributed to the vibration of the carbonyl (ν(CO)) stretch mode in BAD [40]. The stretching vibrations of C-H in BAD were found at 2853 cm^−1^. The peaks in the range of 2900 to 3130 cm^−1^, which gradually increased with reaction time, were assigned to HB, indicating the generation of more HB. The intense bands at 1452 and 1495 cm^−1^ were associated with aromatic δ(C-C) and ν(C−H) modes [40], which can be assigned to HB. Moreover, the peaks at 1583 cm^−1^ and 1601 cm^−1^ were attributed to the C=C skeleton vibration of mononuclear aromatic hydrocarbons, and the peak at 3062 cm^−1^ was assigned to C-H stretching vibration on the benzene ring of HB. These findings provide valuable information on the reaction mechanism and intermediates involved in the photocatalytic coupled system of CO_2_ reduction and benzyl alcohol oxidation over the ZIS catalysts.

## 3. Materials and Methods

### 3.1. Preparation of Zn_m_In_2_S_3+m_ Photocatalysts

Zn_m_In_2_S_3+m_ samples were synthesized by a simple hydrothermal method. For each preparation, 0.8 mmol ZnSO_4_·4H_2_O, 1.6 mmol InCl_3_·4H_2_O, 0.65 g CTAB, and 7 mmol thioacetamide (TAA) were dispersed into 70 mL deionized water. After stirring for 60 min, the above suspension was transferred into a 100 mL Teflon-lined autoclave (Anhui Kemi Machinery Technology Co., Ltd., Hefei, China) and heated at 160 °C for 12 h. After natural cooling to room temperature, the precipitate was washed with absolute ethanol and deionized water several times and dried at 60 °C in a vacuum oven. The as-obtained samples, ZnIn_2_S_4_, Zn_2_In_2_S_5_, Zn_3_In_2_S_6_, Zn_4_In_2_S_7_, and Zn_5_In_2_S_8_ were defined as ZIS1, ZIS2, ZIS3, ZIS4, and ZIS5, respectively.

### 3.2. Characterizations

The structures of samples were analyzed by powder X-ray diffraction (XRD) using a Bruker D8 advance X-ray diffractometer (Karlsruhe, Germany) with Cu Kα radiation (λ = 0.1540 nm) and a scanning speed of 3· min^−1^. Fourier transform infrared spectroscopy (FT-IR) was measured on an iS50 (Waltham, MA, USA). The optical properties were characterized by UV-Vis diffuse reflectance spectroscopy (UV-Vis DRS) using a UV-Vis spectrophotometer (Shimadzu UV-3600, Kyoto, Japan). N_2_ physisorption measurements were carried out at 77 K using a Micromeritics Tristar II 3020 surface area analyzer. Multipoint Brunauer-Emmett-Teller (BET) specific surface areas were then determined from the adsorption isotherms (Micromeritics ASAP 2460, Norcross, GA, USA). The X-ray photoelectron spectroscopy (XPS) was measured on a Thermo Fischer ESCALAB Xi^+^ spectrometer with an Al Kα X-ray beam (Waltham, MA, USA). The binding energies were corrected regarding the C 1s peak of the surface adventitious carbon at 284.8 eV. Transmission electron microscopy (TEM) and high-resolution transmission electron microscopy (HRTEM) images were performed on a JEM-2100 with an accelerating voltage of 200 kV (Akishima-shi, Japan). The morphologies of the photocatalysts were carried out by scanning electron microscope (SEM, Regulus 8200, Tokyo, Japan).

### 3.3. Photoelectrochemical Measurements

Photoelectrochemical measurements: A 5 mg sample was dispersed in 400 μL of deionized water by sonication to obtain a uniform slurry. Then, 20 μL of slurry was deposited as a film on a 0.5 cm × 0.5 cm fluorine-doped tin oxide (FTO) conducting glass to obtain the working electrode. After drying at room temperature, the working electrode was obtained. Ag/AgCl was used as a reference electrode, and platinum wire as a counter electrode. The photocurrent test and flat band potential (M-S plots) were carried out in a three-electrode system in a 0.2 mol L^−1^ Na_2_SO_4_ solution. Electrochemical impedance spectra (EIS) are carried out in a mixture of 0.1 mol L^−1^ KCl and 0.1 mol L^−1^ K_3_[Fe(CN)_6_]/K_4[_Fe(CN)_6_].

### 3.4. Photocatalytic Activity Testing

The coupling activity of photocatalytic CO_2_ reduction and benzyl alcohol oxidation was tested in a visible high-temperature and high-pressure reactor. Typically, 5 mg photocatalyst and 5 mL acetonitrile containing 5 mM benzyl alcohol and 0.1 g K_2_CO_3_ were added into the reactor, which was then ultrasonicated to ensure even dispersion of the catalyst. The reactor was then vacuumed to remove air and finally was stirred in the dark for 30 min to achieve a dynamic dissolution equilibrium of CO_2_ in an atmosphere of CO_2_ gas. The Xe lamp (λ > 420 nm) was used to provide the light source for the reaction. Gas products were collected using a gas injection needle with a quantitative ring, and CO and other products were detected by a flame ionization detector (FID), while H_2_ was detected by a thermal conductivity detector (TCD) gas chromatograph. The liquid products were collected, diluted with acetonitrile, and detected by high-performance liquid chromatography (HPLC).

### 3.5. DFT Calculations

The theoretical simulations were conducted via the Materials Studio (BIOVIA V2017, San Diego, CA, USA) equipped with the CASTEP mode. Also, we utilized the Perdew–Burke–Ernzerhof (PBE) form exchange-correlation functional within the generalized gradient approximation (GGA). The structures of the (001) plane of ZIS1 and ZIS4 were optimized. The formation energy barrier of H_2_ and CO was conducted through the Vienna ab initio Simulation Package (VASP) with the projector augment wave method [41]. A generalized gradient approximation of the Perdew–Burke–Ernzerhof (PBE) functional was used as the exchange-correlation functional. The Brillouin zone was sampled with 2 × 2 × 1 K points for the surface calculation. The cutoff energy was set as 500 eV, and structure relaxation was performed until the convergence criteria of energy and force reached 1 × 10^−5^ eV and 0.02 eV Å^−1^, respectively. A vacuum layer of 15 Å was constructed to eliminate interactions between periodic structures of the surface models. The van der Waals interaction was amended by the zero damping DFT-D3 method of Grimme [42]. The Gibbs free energy was calculated as ΔG = ΔE + ΔEZPE − TΔS, where the ΔE, ΔEZPE, and ΔS are electronic energy, zero-point energy, and entropy difference between products and reactants. The zero-point energies of isolated and absorbed intermediate products were calculated from the frequency analysis. The vibrational frequencies and entropies of molecules in the gas phase were obtained from the National Institute of Standards and Technology (NIST) database [43].

## 4. Conclusions

In summary, this study investigated the effects of composition faults in Zn_m_In_2_S_3+m_ on simultaneous photocatalytic CO_2_ reduction and selective BA oxidation. By adjusting the element composition, the band gap energy (Eg) of ZnmIn_2_S_m+3_ could be controlled, resulting in adjustable redox ability. The CO_2_ reduction activity and selectivity of BA oxidation products were found to be influenced by the Zn/In ratio in ZIS. Specifically, ZIS4 exhibited higher CO_2_ adsorption capacity and lower CO_2_ activation, while ZIS1 had a higher energy barrier for H_2_ evolution. The presence of both a CO_2_ atmosphere and a BA solution was crucial for achieving a high generation rate of CO and C-C coupling products. Moreover, the formation rate of BAD decreased with increasing Zn/In ratio, while the formation rates of hydrogenation products, HB and BZ, increased and reached their highest values at ZIS3. The selectivity of BAD decreased from ZIS1 to ZIS4, while the selectivity of HB increased. The yield of BAD decreased, while the yield of HB and BZ increased with increasing Zn/In ratio. These results highlight the potential of adjusting both the H_2_/CO ratio and the oxidation products of BA by altering the Zn/In ratio in ZIS. Overall, this study provides valuable insights into the role of the Zn/In ratio in the simultaneous photocatalytic CO_2_ reduction and selective BA oxidation process.

## Data Availability

Not applicable.

## Supplementary Materials

The following supporting information can be downloaded at: https://www.mdpi.com/article/10.3390/molecules28186553/s1. Figure S1. Macro images of ZIS samples. Figure S2. The band gap of (a) ZIS1 and (b) ZIS4 by DFT calculation. Figure S3. CO2 adsorption-desorption isotherms of ZIS1 and ZIS4 samples. Figure S4. The HPLC spectra of (a) BAD, (c) HB, (e) BZ and (g) PHE; the corresponding standard curves of (b) BAD, (d) HB, (f) BZ and (h) PHE. Figure S5. The HPLC spectra of products generated from (a) ZIS1 and (b) ZIS4. Figure S6. Controlling experiments over ZIS4 sample. (a) generation rate of H2 and CO, (b) generation rate, (c) selectivity and (d) yield of products form BA oxidation.Figure S7. XRD patterns of ZIS4 before and after reaction. Figure S8. SEM image of ZIS4 after reaction.

## Sample Availability

Samples of the ZIS are available from the authors.
