# Supplementary material for: Photoredox Coupling of CO2 Reduction with Benzyl Alcohol Oxidation over Ternary Metal Chalcogenides (ZnmIn2S3+m, m = 1–5) with Regulable Products Selectivity
Source: Molecules. 2023 Sep 10;28(18):6553. doi: 10.3390/molecules28186553 (PMC10537807; doi:10.3390/molecules28186553)
Supplement: Supplementary file 1 [file molecules-28-06553-s001.zip › molecules-2536487-supplementary.pdf]

Supporting Information for

Photoredox coupling of CO_2 reduction with benzyl alcohol oxidation over ternary metal chalcogenides ($Zn_mIn_2S_{3+m}$, m = 1–5) with regulable products selectivity

Zisheng Du[1], Kexin Gong[2], Zhiruo Yu[1], Yang Yang*[1,2,3], Peixian Wang[4], Xiuzhen Zheng[1], Zhongliao Wang[1], Sujuan Zhang[1], Shifu Chen[1,2], and Sugang Meng*[1,2,4]

[1] Key Laboratory of Green and Precise Synthetic Chemistry and applications, Ministry of Education, College of Chemistry and Materials Science, Huaibei Normal University, Huaibei, 235000, P. R. China.

[2] Key Laboratory of Pollutant Sensitive Materials and Environmental Remediation, Key Lab of Clean Energy and Green Circulation, Huaibei Normal University, Huaibei 235000, P. R. China.

[3] Shanghai Key Laboratory of Atmospheric Particle Pollution and Prevention (LAP[3]), Fudan University, Shanghai 200438, P. R. China.

[4] State Key Laboratory Incubation Base for Green Processing of Chemical Engineering, School of Chemistry and Chemical Engineering, Shihezi, 832003, P. R. China.

* Corresponding authors, E-mails: yangy@chnu.edu.cn (Y. Yang); mengsugang@126.com (S. Meng, Tel: +86-0561-3802235).

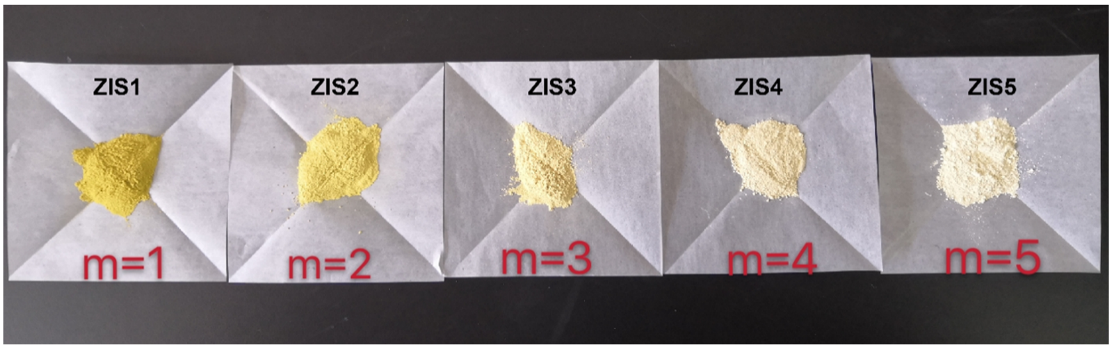

Figure S1. Macro images of ZIS samples.

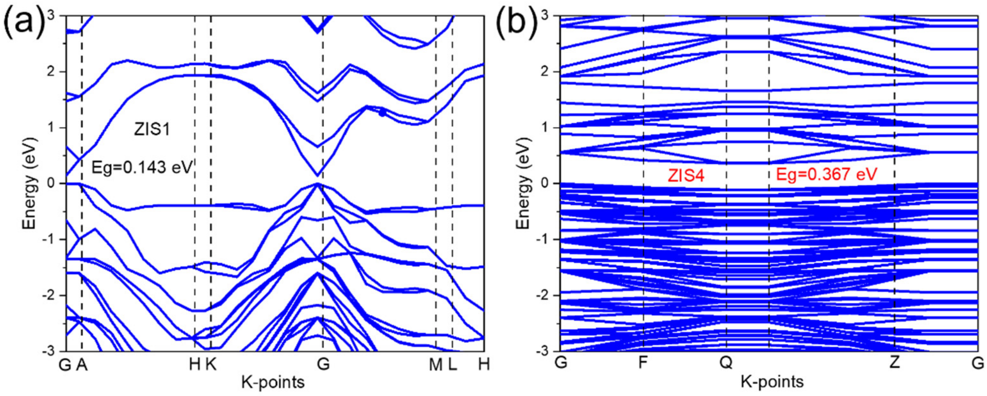

Figure S2. The band gap of (a) ZIS1 and (b) ZIS4 by DFT calculation.

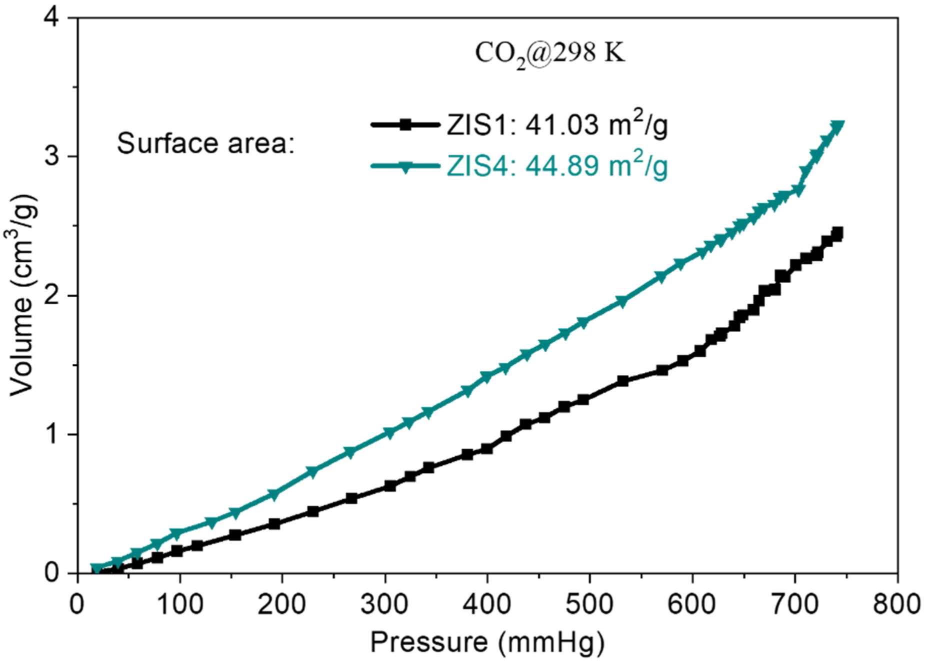

Figure S3. CO_2 adsorption-desorption isotherms of ZIS1 and ZIS4 samples.

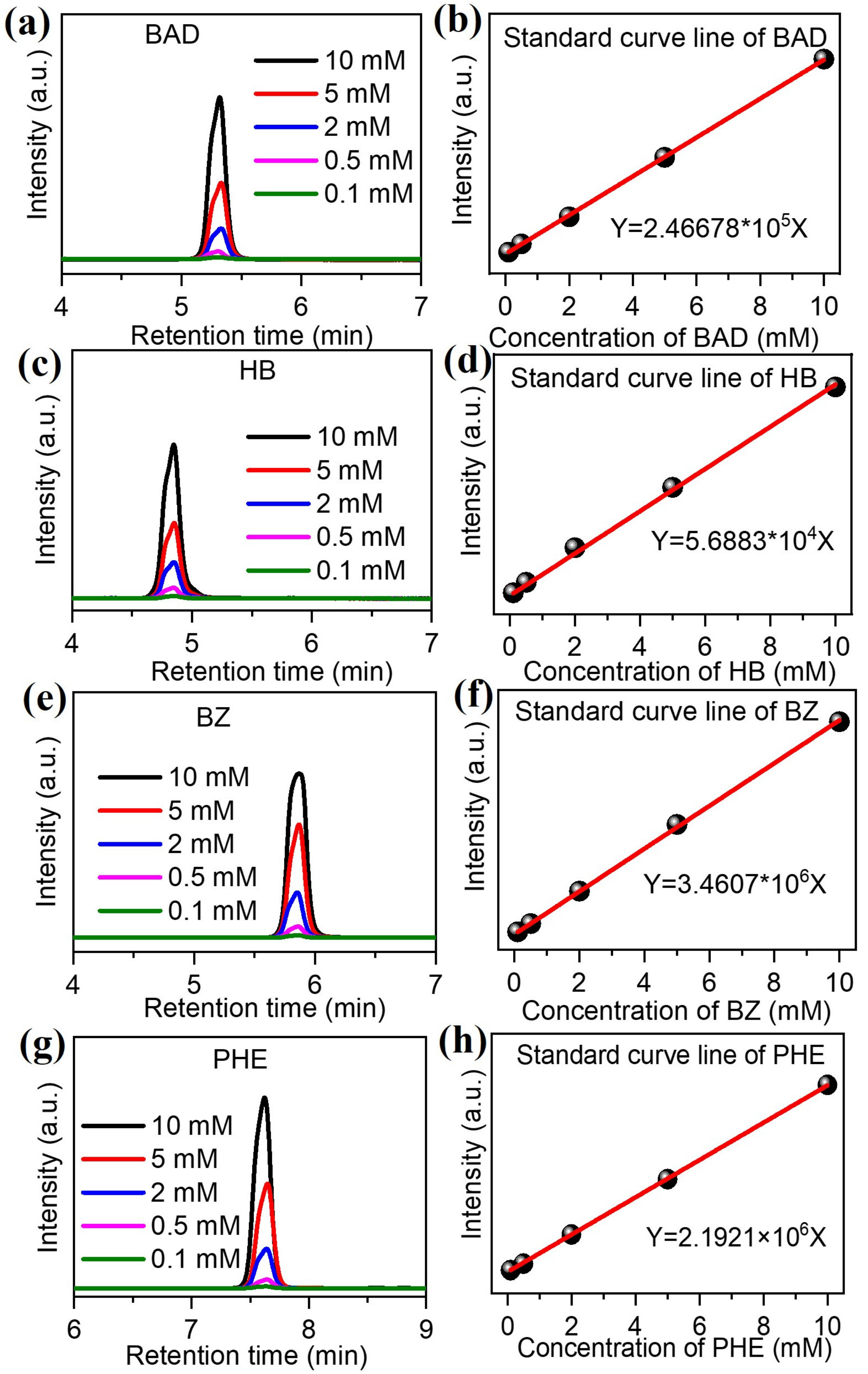

Figure S4. The HPLC spectra of (a) BAD, (c) HB, (d) BZ and (e) PHE; the corresponding standard curves of (b) BAD, (d) HB, (e) BZ and (h) PHE.

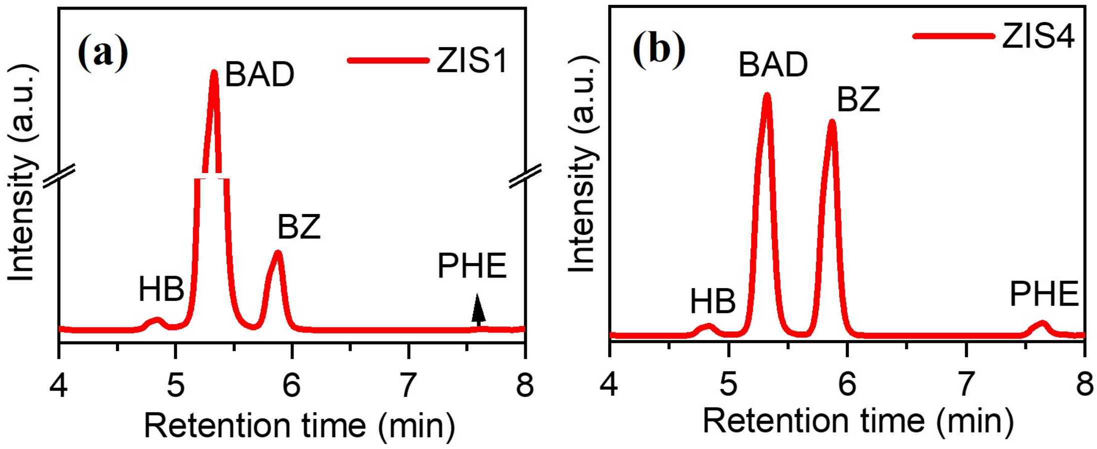

Figure S5. The HPLC spectra of products generated from (a) ZIS1 and (b) ZIS4.

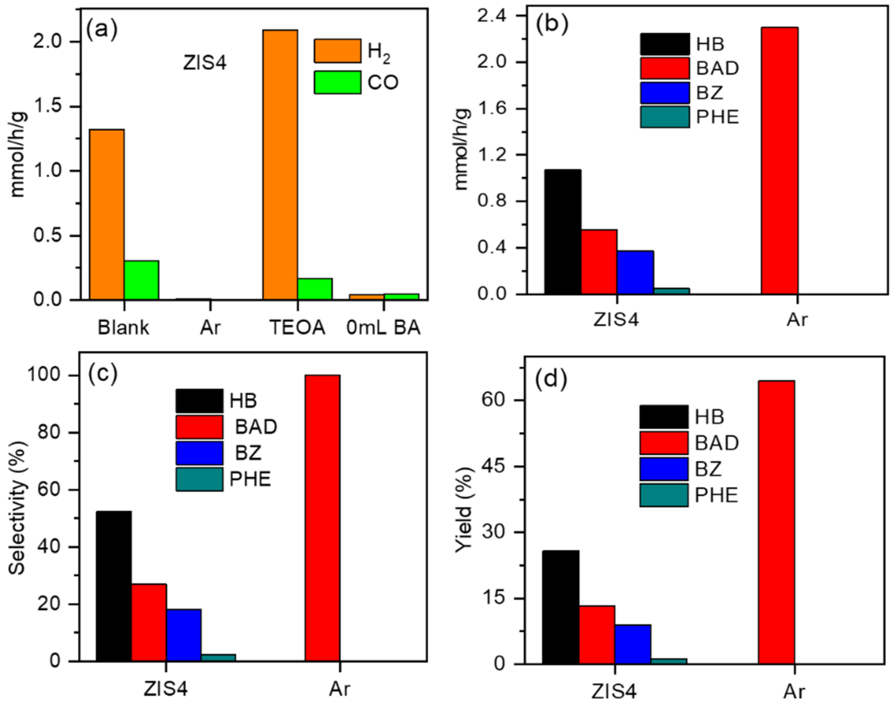

Figure S6. Controlling experiments over ZIS4 sample. (a) generation rate of H_2 and CO, (b) generation rate, (c) selectivity and (d) yield of products form BA oxidation.

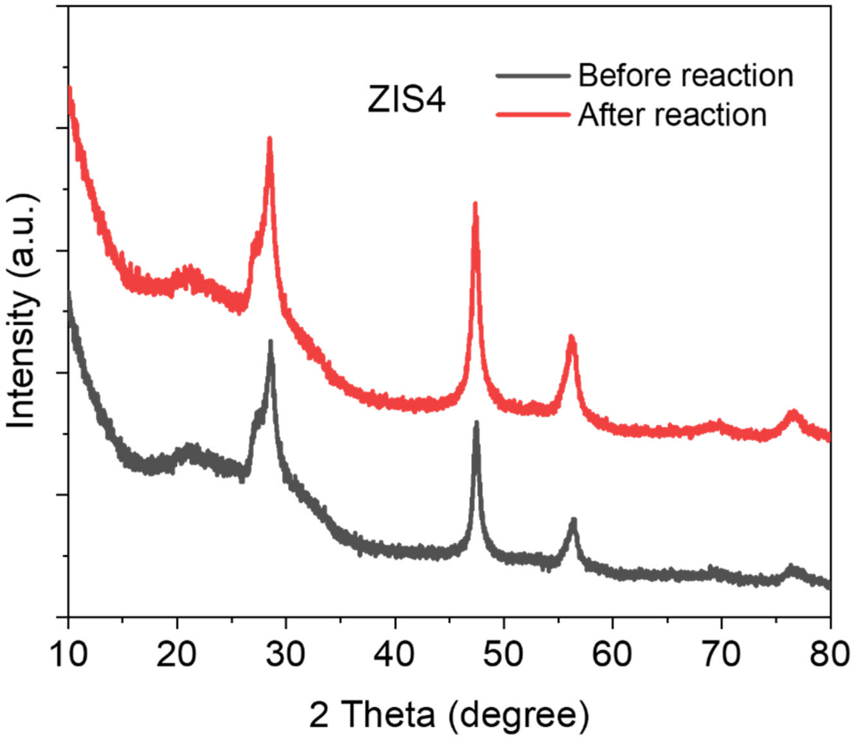

Figure S7. XRD patterns of ZIS4 before and after reaction.

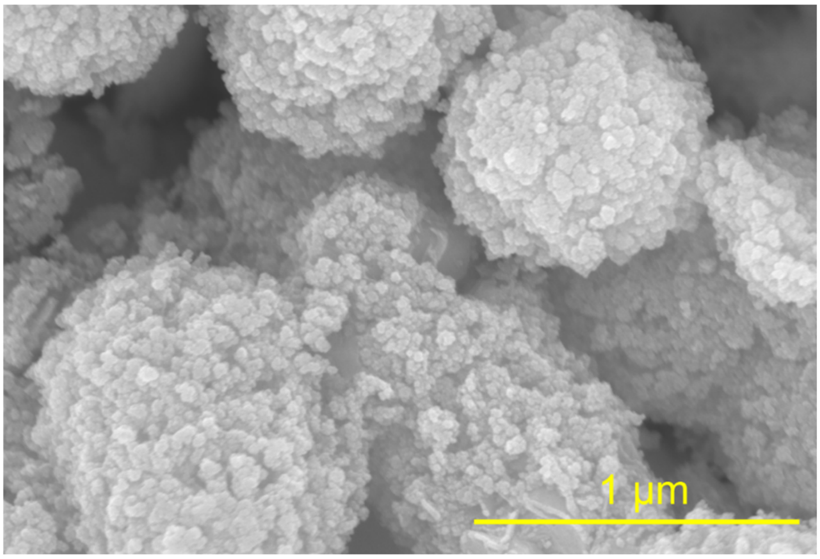

Figure S8. SEM image of ZIS4 after reaction.
